# Fungi-based treatment of real brewery waste streams and its effects on water quality

**DOI:** 10.1007/s00449-019-02130-9

**Published:** 2019-04-25

**Authors:** M. Hultberg, H. Bodin

**Affiliations:** 1 Department of Biosystems and Technology, Swedish University of Agricultural Sciences, P.O. Box 103, 230 53 Alnarp, Sweden; 2 Division of Natural Sciences, Kristianstad University, Kristianstad, Sweden

**Keywords:** Micro-brewery, *Pleurotus ostreatus*, Submerged fungal cultivation, *Trichoderma harzianum*, Water treatment

## Abstract

Nutrient-rich liquid waste streams generated during the beer brewing were treated by submerged fungal growth. Among five filamentous fungal strains tested, *Pleurotus ostreatus* and *Trichoderma harzianum* were selected for treatment of run-off from spent grain and hot trub, respectively. In both waste streams, nitrogen was well removed by fungal treatment, with a maximum reduction of 91.5 ± 0.5% of total nitrogen in run-off from spent grain treated with *P. ostreatus* and 77.0 ± 3.1% in hot trub treated with *T. harzianum*. Removal of phosphorus was considerably lower, with maximum removal of total phosphorus of 30.8 ± 11.1% for the *P. ostreatus* treatment and 16.6 ± 7.8% for the *T. harzianum* treatment. Considering the high concentration of phosphorus in the waste sources (320–600 mg L^−1^), additional techniques for its removal are needed. In the *P. ostreatus* treatment, a total amount of 13.2 ± 2.2 g L^−1^ dwt of biomass with a protein concentration of 11.6 ± 2.1% was produced.

## Introduction

Beer production is a significant contributor to the economy in many countries world-wide [1]. Moreover, in the past decade there has been an impressive upturn in micro-breweries and this development has resulted in an increase in brewery waste streams [2]. Typical liquid waste streams generated during the brewing process are run-off from spent grain from malt mashing and boiling, hot trub from hot wort tank boiling and fermentation slurry [3].

Breweries generally mix all liquid waste streams, including water from tank washing, and discharge them as combined brewery wastewater, generating 3–20 units of wastewater per unit of beer produced [1, 4]. Levels of the most important water quality parameters in combined brewery wastewater are reported to be 2000–6000 mg chemical oxygen demand (COD) L^−1^, 25–80 mg total Kjeldahl nitrogen L^−1^ and 10–50 mg phosphates L^−1^ [1, 3, 4]. The waste streams hot trub and fermentation slurry represent around 3% of the total wastewater volume, but 97% of the load of organic carbon (C) [1]. Thus, the high nutrient levels in the combined wastewater are due to extremely high nutrient concentrations in certain waste streams produced during the brewing process.

Brewery wastewater is generally discharged to municipal wastewater treatment plants or directly into water bodies such as rivers, lakes or the ocean in areas with poor environmental legislation [1, 4]. Waste management in the beer brewing industry is usually a substantial cost factor, especially for micro-breweries, due to sewer discharge fees imposed on effluent volumes and, more often, on organic loads [1]. Breweries discharging to municipal wastewater treatment plants are frequently required to carry out pre-treatment to meet legislation and reduce the load on the treatment plant. Breweries that have their own wastewater treatment, which usually comprises sedimentation and energy-intensive aerobic treatment, are faced with several problems, such as failure to meet water quality standards set by local authorities and/or large amounts of low-value sludge, which is costly to dispose of [1, 5].

Since waste streams from brewing processes have high biodegradability, biological treatment is frequently used [4]. In addition, production of by-products from brewery waste streams is possible, since the streams contain high-quality nutrients without problematic pollutants such as pharmaceuticals and enteric pathogens [6]. One option for microbial biomass production is submerged cultivation of filamentous fungi. However, this technique has received less attention in the past three decades for treatment of waste streams, possibly due to established use of bacteria-based treatment as the standard [7]. Nevertheless, fungi-based treatment of brewery waste streams could offer advantages over bacteria-based wastewater treatment techniques. One benefit is that the growing mycelium converts organics and nutrients in the waste stream into easily dewaterable fungal biomass, with uses as, e.g. a food/feed ingredient or a component in biomaterial, while simultaneously reducing the organic load in contrast to low-value sludge produced during bacterial treatment [5, 7].

In a previous study, we examined fungi-based treatment of combined synthetic brewery waste streams [8]. However, an interesting and resource-efficient approach would be to keep the waste streams from the different brewing processes separate and apply an optimised treatment to each specific waste stream. Thus, the aim of this study was to investigate submerged cultivation of filamentous fungi as a treatment method for separated nutrient-rich waste streams from micro-breweries. The potential for nutrient reduction by the fungi-based treatment was evaluated against official Swedish water quality parameters established for small-scale wastewater treatment systems [9]. The potential for production of fungal biomass in two different waste streams was also evaluated and the quality of the biomass produced, including total protein and amino acid composition, was determined for one selected treatment. The fungal species used in the study were either edible or have a long record of safe use.

## Materials and methods

### Microorganisms

Five fungal species were used in the study: *Agaricus bisporus* M7215, *Lentinula edodes* M3782, *Pleurotus ostreatus* M2140, *Trichoderma harzianum* CBS 226.95 and *Trametes versicolor* M9912. These fungal strains were purchased from the American Type Culture Collection (ATCC), Mycelium BVBA, Belgium (M) and the CBS Fungal Biodiversity Centre, The Netherlands (CBS). The species *A. bisporus*, *P. ostreatus* and *L. edodes* were selected for the experiments since they are well-known edible mushrooms. *T. versicolor* was included since it is an edible, however considered unpalatable, and fast-growing species with biotechnological and pharmaceutical application [10]. *T. harzianum* was included since this species has a long record of large-scale use in agriculture [11].

For long-term storage the strains were kept on malt agar (MA), spiked with streptomycin in a concentration of 100 µg mL^−1^ to minimise bacterial contamination. Fungal inoculum was produced by incubating the strains at 27 °C on Petri dishes containing 20 mL of potato dextrose agar (PDA). The cultivation period was 10 days, but *A. bisporus* grew more slowly than the other strains and was cultivated for 20 days. Circular slants (diameter 15 mm) were cut from the PDA plates and used as fungal inoculum in all experiments.

### Brewery wastewater

Three different brewery waste streams (spent grain, hot trub, fermentation slurry) were collected from a local micro-brewery (Lundabryggeriet, Torna Hällestad, Sweden). The spent grain was immediately placed in a cloth and the run-off was collected. Due to high viscosity, the filtered run-off from spent grain was diluted with an equal amount of sterile distilled water. After this step, each waste stream was filtered through 0.5 mm mesh size stainless steel drum sieves (AB Åsbrink & Co Malmö). The filtered waste streams were autoclaved and kept at 4 °C until the start of the experiments. The pH and concentrations of COD, total nitrogen (TN), ammonium-nitrogen (NH_4_^+^-N) and total phosphorus (TP) in the waste streams before the start of the experiments are presented in Table 1.

**Table 1:** Initial pH and concentration (mean ± SD) of the water quality parameters chemical oxygen demand, total nitrogen, ammonium-nitrogen and total phosphorus in spent grain run-off, hot trub and fermentation slurry waste streams collected from a micro-brewery, *n* = 3

Parameter	Spent grain run-off	Hot trub	Fermentation slurry
pH	5.1 ± 0.0	5.1 ± 0.0	4.7 ± 0.0
COD (mg L^−1^)	51,525 ± 10,004	126,366 ± 15,381	213,661 ± 19,202
Total nitrogen (mg L^−1^)	730 ± 48.1	451 ± 21.2	2291 ± 413
Ammonium-nitrogen (mg L^−1^)	21.3 ± 1.8	35.4 ± 0.8	158 ± 18.7
Total phosphorus (mg L^−1^)	367 ± 18.1	320 ± 18.2	601 ± 30.3

### Experimental setup

All experimental setups involved batch reactors in Erlenmeyer glass flasks on a horizontal orbital shaker (VWR, Advanced 5000 shaker) at 150 rpm at 27 °C. Initial dry weight of the inoculum (mycelium and PDA) was determined for each strain by drying PDA slants with inoculated mycelium at 60 °C until constant weight. Control treatments containing PDA slants without fungal mycelium were also included.

The first experiment comprised five reactor treatments on 30-mL samples of each of the autoclaved waste streams, using the five fungal strains. The fungal strains that gave the highest dry weight biomass production and greatest COD reduction in selected waste streams after 7 days were used for a second experiment, in which samples were taken on day 0, 3, 6, 8 and 10 for estimation of nutrient reduction over time. In the second experiment, each reactor contained 200 mL of the waste stream.

### Analysis

#### Fungal biomass production

In both the first and second experiment, fungal biomass was collected by filtration using pre-dried and pre-weighed nylon filters (mesh size 0.6 mm) and was washed twice with an equal amount of distilled water. In the first experiment the biomass was collected on day 7 and in the second experiment the biomass was collected on day 10. To determine the total dry weight of the collected fungal biomass after filtration, the filters were dried at 60 °C to constant weight. The final amount of fungal biomass produced for each fungus was determined as the difference between total dry weight biomass at the end of the experiment and initial dry weight of the inoculum.

However, when *T. harzianum* CBS 226.95 was cultured in hot trub in the first experiment, dispersed mycelial growth [12] and increased viscosity of the fluid made filtration difficult. In order to determine biomass production of *T. harzianum* CBS 226.95 on day 10 in the second experiment, filtration was therefore replaced with centrifugation (1000 g, 5 min) and an equal volume of non-inoculated hot trub was used as a control.

#### Protein concentration and amino acid composition in selected biomass

As the edible fungi *P. ostreatus* M2140 (oyster mushroom) was included in second experiment, total protein and amino acid composition of fungal biomass produced was also determined. Total amount of protein was analysed by the Dumas method [13], using a Thermo Scientific™ FLASH 2000 CHNS/O Analyzer and a conversion factor of 4.38 for total nitrogen [14]. Amino acid composition (alanine, arginine, aspartic acid, cysteine, glutamic acid, glycine, histidine, isoleucine, leucine, lysine, methionine, phenylalanine, proline, serine, threonine, tyrosine and valine) was determined at a certified laboratory (Eurofins Food & Agro Testing Sweden AB, Linköping, Sweden) by ion exchange chromatography according to the method by Llames and Fontaine [15].

#### Analysis of nutrient reduction in the waste streams

Nutrients in the waste streams were analysed before and after treatment. The initial concentrations are as presented in Table 1. For nutrient analysis after treatment, the biomass was separated by filtration as described above. Sterile treatment of each waste stream, incubated under the same conditions as the fungal treatments, was included as a control. In the first experiment, COD was determined at the end of the experiment on day 7. In the second experiment, TN, NH_4_^+^-N, TP and COD were analysed in samples taken over time, with the final sampling at the end of the experiment on day 10. Concentration of TN was determined with Hach Lange LCK 338 (EN ISO 11905-1), NH_4_^+^-N with Hach Lange LCK 303 (ISO 7150-1), TP with Hach Lange LCK 350 (EN ISO 6878) and LCK 349 (EN ISO 6878), and COD with Hach Lange LCK 014 (ISO 6060-1989). These analyses were performed in room temperature (20–22 °C).

### Data analysis and statistics

The relative reduction in nutrients in the selected waste streams was calculated as:

$${\text{Relative}}\,{\text{reduction }} = \left( {\frac{{(C_{\text{initial}} - C_{\text{final}} )}}{{C_{\text{initial}} }}} \right) \times 100,$$

where *C*_initial_ is initial nutrient concentration and *C*_final_ is final nutrient concentration after fungal treatment (both in mg L^−1^).

All treatments were carried out in triplicate and statistical analyses were performed using Minitab version 17 for Windows. The data obtained were analysed by analysis of variance followed by Tukey’s multiple comparison test, and correlations were analysed by Pearson correlation coefficient (*r*), with *p *< 0.05 set as the level of significance.

## Results

### Run-off from spent grain

Results from all fungal treatments showed a significant positive correlation between the amount of fungal biomass produced and the COD removed in run-off from spent grain (*r* = 0.89). All fungal treatments on run-off from spent grain, except *A. bisporu*s, resulted in a significant biomass increase compared with the sterile control treatment. Treatment of run-off from spent grain using *P. ostreatus*, *T. harzianum* and *T. versicolor* resulted in significantly higher biomass production than treatment using *L. edodes* (Table 2). In addition, those three treatments achieved a significantly greater COD reduction than the other treatments. There was no significant difference between *P. ostreatus*, *T. harzianum* and *T. versicolor* as regards biomass production and COD removal. *Pleurotus ostreatus* was selected for further studies in run-off from spent grain in the second experiment.

**Table 2:** Amount of biomass produced (g L^−1^, dwt) and relative reduction in chemical oxygen demand (mean ± SD) compared with initial values obtained after 7 days of fungal growth in spent grain run-off, hot trub and fermentation slurry waste streams collected from a micro-brewery, *n* = 3

Fungal strain	Run-off spent grain		Hot trub		Fermentation slurry	
	Biomass	COD (%)	Biomass	COD (%)	Biomass	COD (%)
*A. bisporus*	1.3 ± 0.2a*	37.8 ± 3.4a	2.1 ± 0.8a	21.3 ± 7.4ab	2.1 ± 0.5a	9.4 ± 2.0a
*L. edodes*	9.0 ± 0.7b	49.1 ± 4.0a	5.5 ± 0.2ab	11.9 ± 11.2b	2.0 ± 0.2a	7.0 ± 1.5a
*P. ostreatus*	22.7 ± 2.0c	72.2 ± 5.3b	6.0 ± 0.9ab	35.8 ± 3.1a	2.2 ± 0.2a	4.0 ± 3.1a
*T. harzianum*	17.3 ± 1.4c	84.9 ± 1.3b	15.2 ± 3.1c	61.4 ± 4.4c	4.1 ± 0.7b	19.6 ± 5.5b
*T. versicolor*	16.4 ± 5.3c	77.4 ± 8.2b	9.8 ± 1.6b	36.0 ± 7.3a	1.5 ± 0.2a	6.0 ± 1.9a

* Values within columns followed by different letters are significantly different (*P* < 0.05, Tukey’s test)

### Hot trub

There was a significant positive correlation between the amount of fungal biomass produced and the COD removed in hot trub (*r* = 0.82). Treatment of hot trub with *T. harzianum* or *T. versicolor* resulted in the highest biomass production and comparisons between treatments showed that treatment with *T. harzianum* resulted in significantly higher production of biomass compared with *T. versicolor* (Table 2). A significant reduction in COD concentration compared with the other treatments was observed in the treatment with *T. harzianum*. Thus, *Trichoderma harzianum* was selected for further studies with hot trub in the second experiment.

### Fermentation slurry

There was no correlation between the amount of fungal biomass produced and the COD removed in fermentation slurry (*r* = 0.33). For fermentation slurry, treatment with *T. harzianum* resulted in a significantly higher amount of biomass and reduction of COD compared with the other treatments (Table 2). Submerged fungal growth for treatment of fermentation slurry was not pursued further in this study.

### Treatment of run-off from spent grain using *P. ostreatus*

**Fig. 1:**
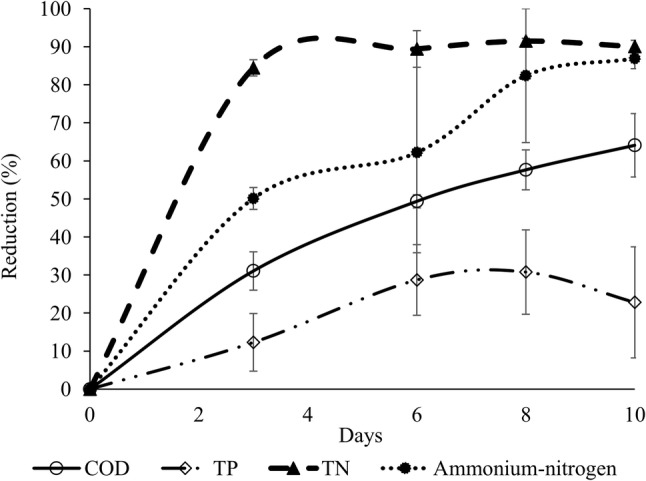
Relative reduction compared with initial concentrations of water quality parameters in run-off from spent grain treated using submerged growth of the fungus *Pleurotus ostreatu*s. *COD* chemical oxygen demand, *TP* total phosphorus, *TN* total nitrogen

Treatment of spent grain with *P. ostreatus* gave significantly lower concentrations of NH_4_^+^-N and TN compared with the control treatment on day 3, and of all water quality parameters on days 6–10 (Fig. 1). On day 10, this fungal treatment had a TN concentration of 72.8 ± 12.1 mg L^−1^ while the control treatment had a concentration of 656 ± 156 mg L^−1^. For TP, treatment with *P. ostreatus* gave a concentration of 284 ± 53.6 mg L^−1^ on day 10, while the control treatment had a concentration of 457 ± 92.8 mg L^−1^. Removal of COD also increased over time in this fungal treatment and the remaining concentration on day 10 was 18,503 ± 4294 mg L^−1^, compared with 53,862 ± 9622 mg L^−1^ in the control treatment. The pH in the run-off from spent grain increased slightly in the *P. ostreatus* treatment, reaching a value of 5.7 ± 0.1 on day 10, compared with 5.2 ± 0.0 in the control treatment.

The total amount of biomass composed of mycelium of *P. ostreatus* produced after 10 days of growth was 13.2 ± 2.2 g L^−1^ dwt. No biomass production was observed in the control treatment. The collected biomass from the *P. ostreatus* treatment had a total protein concentration of 11.6 ± 2.1%. The amino acid composition was dominated by glutamic acid, aspartic acid and proline (Table 3).

**Table 3:** Amino acid composition (g 100 g^−1^ dry weight) of mycelium of *Pleurotus ostreatus* (mean ± SD) cultivated in run-off from spent grain

Amino acid	*P. ostreatus*	Range of mean values (OECD 2013)
Alanine	0.85 ± 0.06	0.95–2.86
Arginine	0.76 ± 0.04	0.95–2.76
Aspartic acid	1.16 ± 0.03	1.42–3.66
Cysteine	0.24 ± 0.02	0.12–0.38
Glutamic acid	1.93 ± 0.17	2.71–5.84
Glycine	0.64 ± 0.02	0.70–1.71
Histidine	0.35 ± 0.02	0.31–1.24
Isoleucine	0.52 ± 0.01	0.71–1.62
Leucine	0.92 ± 0.02	1.18–2.57
Lysine	0.69 ± 0.01	1.10–2.29
Methionine	0.22 ± 0.01	0.26–0.44
Phenylalanine	0.57 ± 0.01	0.66–1.52
Proline	1.05 ± 0.05	0.39–1.52
Serine	0.65 ± 0.01	0.72–1.81
Threonine	0.60 ± 0.01	0.73–1.71
Tyrosine	0.37 ± 0.02	0.54–2.74
Valine	0.67 ± 0.02	0.77–2.10

The range of mean values for each amino acid according to OECD [15] is also shown

### Treatment of hot trub using *T. harzianum*

**Fig. 2:**
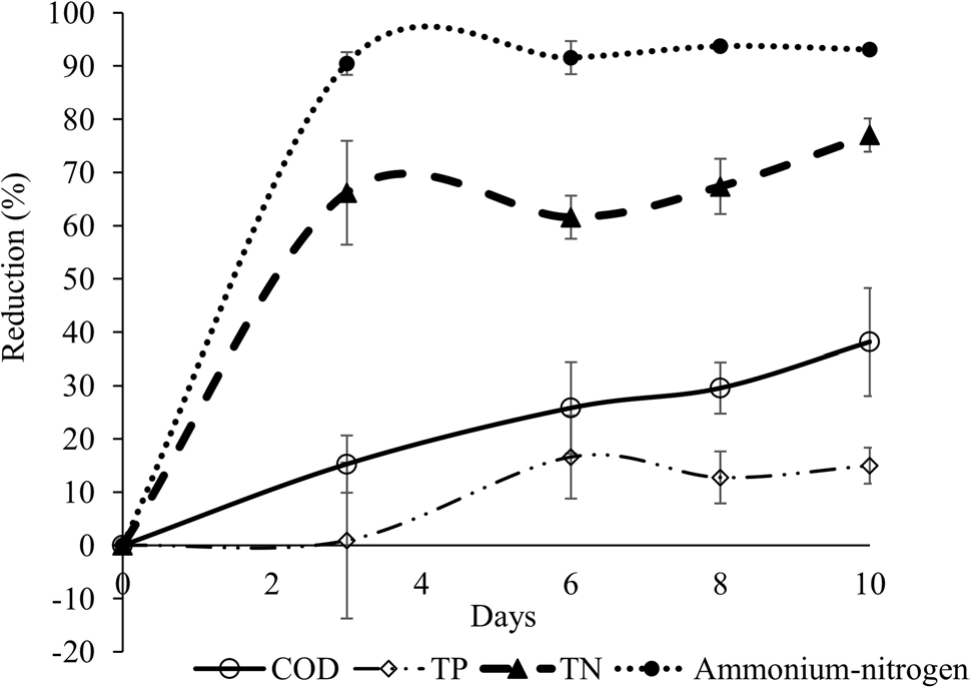
Relative reduction compared with initial concentrations of water quality parameters in hot trub treated using submerged growth of *Trichoderma harzianum*. *COD* chemical oxygen demand, *TP* total phosphorus, *TN* total nitrogen

Treatment of hot trub with *T. harzianum* resulted in a similar pattern as observed on treating run-off of spent grain with *P. ostreatus*, with significantly lower concentrations of NH_4_^+^-N and TN compared with the control treatment on day 3, and of all parameters on days 6–10 (Fig. 2). On day 10, the fungal treatment had a TN concentration of 104 ± 14.1 mg L^−1^, while the control treatment had a concentration of 292 ± 20.8 mg L^−1^. For TP, the fungal treatment had a concentration of 271 ± 15.3 mg L^−1^ on day 10 while the control treatment had a concentration of 413 ± 27 mg L^−1^. The COD removal increased over time in the fungal treatment and the remaining concentration on day 10 was 78,142 ± 12,778 mg L^−1^, compared with 129,972 ± 11,575 mg L^−1^ in the control treatment.

Similarly to the *P. ostreatus* treatment of run-off from spent grain, the *T. harzianum* treatment resulted in significantly higher pH in hot trub compared with the control treatment throughout the study. On day 10, the pH was 6.1 ± 0.1 in the fungal treatment and 4.9 ± 0.0 in the control treatment.

The total amount of biomass, composed of mycelium of *T. harzianum* and solid particles in the hot trub, produced after 10 days of fungal growth was 40.19 ± 1.02 g L^−1^ dwt. The amount of biomass collected in the sterile control at the end of the experiment was 20.11 ± 1.66 g L^−1^ dwt.

## Discussion

As mentioned, brewery waste is currently mainly treated as a combined and diluted waste stream and there has been little research on separate treatment of the most nutrient-rich waste streams. Of the three liquid waste streams tested in this study, run-off from spent grain and hot trub both supported submerged fungal growth, while less growth was observed in fermentation slurry. The lack of fungal growth in fermentation slurry could potentially be due to residual ethanol or to the ammonium concentration, which was considerably higher than in the other waste streams (Table 1). Dried fermentation slurry has potential for biovalorisation in the food and feed industry [16] and therefore biological treatment of fermentation slurry through submerged fungal growth was not pursued further in this study.

Among the five fungal species tested, *A. bisporus* and *L. edodes* appeared less efficient in treatment of the liquid waste streams. This is in agreement with results obtained when synthetic combined brewery wastewater was treated in a similar experimental setup in our previous study [8]. The fungal species *P. ostreatus*, *T. harzianum* and *T. versicolor* showed better growth and all three strains had high biomass production on run-off from spent grain. As *P. ostreatus*, commonly known as oyster mushroom, has been identified as a potential source of feed nutrients such as essential amino acids [17], this species was selected for further studies on run-off from spent grain.

In this waste stream, the initial ratio of TN to TP was around 2:1 (Table 1). In a study on C:N:P ratio in fungal biomass, variations have been reported between different phyla but on average the stoichiometry is similar to the Redfield values, with an N:P ratio of 16:1 [18]. Thus, for biological treatment the amount of phosphorus was high compared with the amount of nitrogen in run-off from spent grain. This is also reflected in the results, where the relative reductions in TN and NH_4_^+^-N were generally large throughout the study and with a considerably smaller reduction in TP. The maximum reduction in TN and NH_4_^+^-N was 91.5 ± 0.8% and 86.9 ± 2.7%, respectively, whereas the maximum reduction in TP was 30.8 ± 7.8% (Fig. 2). The Swedish Agency for Marine and Water Management [9] has set 50% TN reduction as the legal water quality standard to be achieved by small-scale wastewater treatment systems. Considering the TN reduction results obtained in the present study when using *P. ostreatus* for treatment of run-off from spent grain, the standard for TN was met by a large margin, while the requirement for a TP reduction of 70% was far from being reached.

TP includes all organic and inorganic forms of phosphorus present in the matrix, particulate as well as dissolved. An increase in the control treatment (457 ± 92.8 mg L^−1^ of TP at day 10) compared to initial values (367 ± 18 mg L^−1^ of TP) was observed. The high variability and the increase in the numerical value may be due to the complex matrix. Potentially the presence of particulate phosphorus, which was not dissolved in the hydrolyzing process, was affected over time by abiotic factors.

In Sweden, 90% of the organic carbon in wastewater must be removed in order to meet current regulations on effluent water quality from small-scale wastewater treatment systems [9]. In the first experiment testing fungal treatments, *P. ostreatus*, *T. harzianum* and *T. versicolor* were close to complying with this requirement (Table 2). In the second experiment the reduction in COD increased over time (Fig. 1) and reached its maximum (64.1 ± 8.3%) on day 10. Thus, a treatment period of 10 days was not enough to meet the standard for reduction of organic carbon.

In this context, it is interesting to note that in the first experiment, treatment of run-off from spent grain using *P. ostreatus* resulted in biomass dry weight production of 22.68 ± 2.04 g L^−1^ after 7 days of treatment. In the second experiment, biomass production by the same strain from spent grain was 13.2 ± 2.2 g L^−1^ after 10 days of growth. The lower biomass obtained in the second experiment can most probably be explained by the larger volume of sample in the second experiment, with accompanying difficulties in oxygenation of the medium used for cultivation. In parallel, the first experiment showed a correlation between COD reduction and biomass production for the fungal strains. Thus, optimisation of the cultivation system may lead to increased biomass production, accompanied by a larger COD reduction.

In future development towards a bio-based society, production of microbial proteins based on low-value side-streams can be a powerful asset [19]. The mycelium of *P. ostreatus* produced in the present study had a total protein concentration of 11.6 ± 2.1% of dry weight. This is similar to the average concentration of 13.2% reported by Kalac et al. [20] on reviewing the chemical composition and nutritional value of fruiting bodies of several mushroom species. The amount of individual amino acids in the mycelium produced in run-off from spent grain was generally slightly lower than the mean value reported for fruiting bodies of *P. ostreatus* [17] (see Table 3). Moreover, compared with the amino acid composition in fish meal, a commonly used component to enhance the nutritional properties of feed, the concentration of essential amino acids was low in the harvested mycelium [21]. However, despite these results, pure mycelium of *P. ostreatus* is an interesting by-product and recent research has demonstrated the potential for production and enrichment of vitamin D in the mycelium with UV-irradiation [22].

For hot trub, treatment with *T. harzianum* resulted in high production of biomass and the largest reduction in COD of all treatments (Table 2). This species, a soil fungus with a long tradition of applications within agriculture [11], was therefore selected for further studies in the second experiment. In the hot trub waste stream, the amount of phosphorus was high compared with the amount of nitrogen, with a ratio of TN to TP of around 1.4:1 (Table 1). As observed for treatment of run-off from spent grain with *P. ostreatus*, use of *T. harzianum* to treat hot trub was efficient in reducing TN and NH_4_^+^-N. The 50% TN reduction criterion set by the Swedish Agency for Marine and Water Management [9] was met already on day 3 (Fig. 2). The reduction in TP was considerably lower and reached a maximum of 16.6 ± 7.8%.

Similar to the experiments performed in run-off from spent grain an increase in TP was observed in the sterile control compared to the initial values. As discussed above this may be due to the complex matrix and effects over time on the particulate organic matter. In this waste stream also a decrease in TN was observed over time in the control suggesting gaseous losses of nitrogen.

From the present study, it can be concluded that nitrogen was removed to a large extent by fungal treatment of run-off from spent grain and hot trub, while the removal of phosphorus was considerably lower. Considering the high concentrations of phosphorus in the waste streams and the future need for recycling of this nutrient, additional treatment will be required, e.g. using struvite precipitation [23] or fungal strains specialising in P uptake [24].

In hot trub treated with *T. harzianum*, the reduction in COD increased over time and the maximum reduction (38.2 ± 10.1%) was observed on day 10. Thus, considering the current regulation on effluent water quality from small-scale wastewater treatment systems (requiring 90% reduction in organic carbon), fungal treatment of hot trub was not satisfactory. Zhang et al. [25] reported a more positive result using the related species *Trichoderma viride* to treat winery wastewater, where COD reductions in the range 86–91% after 24 h at a cultivation temperature of 30 °C were achieved. The difference compared with the present study can be explained by the differing chemical composition of the waste stream, with lower COD values in the winery wastewater used by Zhang et al. [25] as a main factor. However, temperature is also an important parameter, as faster growth and higher metabolic rate are observed with increasing temperature.

As previously mentioned, growth of *T. harzianum* in hot trub did not result in production of distinct pellets, but dispersed growth of mycelia and increased viscosity of the waste stream. When the biomass was collected by centrifugation, the amount of biomass produced in the hot trub treated with *T. harzianum* was double that in the control treatment (40.2 ± 1.0 compared with 20.1 ± 1.7 g L^−1^ dwt). This suggests that the amount of fungal biomass produced was approximately 20 g L^−1^ dwt, which is in line with the value obtained in the first experiment (15.2 ± 3.1 g L^−1^). However, the different setups for harvesting makes this comparison very uncertain. In the study by Zhang et al. [25], high biomass production rates (4.50–5.40 g dwt biomass L^−1^ after 24 h of growth) were observed. However, unlike in the treatment using the edible *P. ostreatus*, the uses of the *Trichoderma* biomass produced is less clear. *Trichoderma* sp. has been highlighted as a potential biosorbent [26], and in a longer perspective fungal mycelium may be of use in the production of biomaterial, as exemplified by Haneef et al. [27]. However, from an applied perspective, the use of *T. harzianum* for treatment of hot trub was less successful considering the low reduction in COD and TP and the need for centrifugation for efficient separation of the biomass.

Currently the most common approach by the micro-breweries is to mix the liquid waste streams and discharge them as combined brewery wastewater [1, 4]. However, from an applied perspective it should be pointed out that waste streams are usually handled manually by the micro-breweries. Thus, the most nutrient-rich waste streams can be removed and collected separately. The remaining wastewater would then be less of a burden for the municipal wastewater treatment plant, due to reduction of the organic load, and this approach may also decrease the cost for waste disposal. Our study is performed to develop sustainable use of the separated waste streams and demonstrates that production of fungal biomass, parallel with improved water quality, is possible. However, there are techno-economic limitations of small-scale production of fungal biomass and further research is needed to develop this bio-based technology.
